# Supplementary material for: Nitrogen Dense Distributions of Imidazole Grafted Dipyridyl Polybenzimidazole for a High Temperature Proton Exchange Membrane
Source: Polymers (Basel). 2022 Jun 28;14(13):2621. doi: 10.3390/polym14132621 (PMC9269216; doi:10.3390/polym14132621)
Supplement: Supplementary file 1 [file polymers-14-02621-s001.zip › polymers-1786716-supplementary.pdf]

Supporting Information

Imidazole Grafted Dipyridyl Polybenzimidazole for High Temperature Proton Exchange Membrane

Qi Pei,[a,b]‡ Jianfa Liu,[a,b]‡ Hongchao Li,[a] Wenwen Wang,[a] Jiaqi Ji,[a] Keda Li,[a] Chenliang Gong,*[a] Lei Wang*[b]

[a] State Key Laboratory of Applied Organic Chemistry, Key Laboratory of Special Function Materials and Structure Design of Ministry of Education, College of Chemistry and Chemical Engineering, Lanzhou University, Lanzhou 730000, P. R. China

[b]Shenzhen Key Laboratory of Polymer Science and Technology, College of Materials Science and Engineering, Shenzhen University, Shenzhen 518060, P. R. China

‡ These authors contributed equally to this work.

E-mail address: gongchl@lzu.edu.cn (C. Gong)

wl@szu.edu.cn (L. Wang)

1. Materials and reagents

Poly(2,2′-[p-oxydiphenylene]-5,5′-benzimidazole) (OPBI) powder was purchased from Shanghai Shengjun Polymer Technologies Co., Ltd. 4,4'-Oxybisbenzoic acid, 3,3'-Diaminobenzidine were purchased from TCI (Shanghai) Chemical Industry Development Co., Ltd. [2,2'-Bipyridine]-4,4'-dicarboxylic acid, polyphosphoric acid, $NaHCO_3$, absolute ethyl alcohol (EtOH) and N,N-Dimethylacetamide were purchased from Energy Chemical Co, Ltd. (China). 85 wt.% phosphoric acids were purchased from Shanghai Macklin Biochemical Co., Ltd. 1-(2-chloroethyl) imidazole were purchased from Ark Pharma Scientific Limited (China).

2. Characterization methods

The chemical structures of the DPPBI and grafted ImDPPBI were analyzed using ^{1}H NMR spectroscopy, which was conducted on a Bruker Avance-500 (500MHz) using dimethyl sulfoxide-D_6 (DMSO-d_6) as the solvent. Fourier transform infrared (FT-IR) absorption spectra of COPBI-s were measured at 400 to 4000 cm^{-1} using a Nicolet 6700 spectrometer. A SU-70 SEM was used to examine the surface and cross-section morphologies of the membranes. A TA Q50 thermal gravimetric analyzer was used to determine the weight loss in a dry nitrogen atmosphere with a flow rate of 40 mL min^{-1} and a heating rate of 10 °C min^{-1} from 100 °C to 800 °C.

All membranes were immersed in an 85% phosphoric acid (PA) solution at

160 °C for 24 h. Following that, PA-doped membranes were dried overnight in a vacuum oven to a constant weight. The gross PA uptake was calculated using the following formula:

Gross PA Uptake = $(W_d - W_0)/W_d$ (1)

where W_0 (g) is the weight of the pristine membrane and W_d (g) is the weight of the membrane after saturated PA doping at 160 °C.

Furthermore, phosphoric acid doped content (ADC) is positively correlated with the proton conductivity to some extent. ADC is determined according to the following formula.

ADC = $[W_2 - W_1]/W_1$ (2)

In this equation, W_1 (g) and W_2 (g) represent the weight of the membrane before and after doping, respectively.

The deformation ratio after doping can be calculated from the following quation:

Deformation ratio (%) = $([L_2 \times B_2 \times D_2]/[L_1 \times B_1 \times D_1]) \times 100\%$ (3)

where L_1 (cm), B_1 (cm), and D_1 (mm) represent the length, width, and thickness of the pristine membrane, respectively, whereas L_2 (cm), B_2 (cm), and D_2 (mm) represent the length, width, and thickness of the PA-doped membrane, respectively.

Tensile strength and elongation at break of PA-undoped and -doped membranes (dried overnight in a vacuum oven) were cut into dumbbell-shaped samples and measured using a SANS-CMT4204 system with a 5-mm min^{-1}

extension rate.

The resistance of the PA-doped membranes was tested by an electrochemical workstation (Zahner IM6ex, Germany) using the alternating current impedance method. The parameters are as follows: 1–10^5 Hz frequency, 10 s quiet time, and 0.01 V amplitude. The PA-doped membranes were cut into rectangular samples (1 × 4 cm) and placed on an electrode covered with an airtight mold. The temperature was raised to 120 °C and maintained for 2 h to obtain anhydrous conditions. Then, the mold temperature was raised to 180°C and equilibrated for 15 min. The measurements were taken while the mold was cooled to 120 °C in 10 °C increments, with a 15-min equilibration time for each temperature set point. The proton conductivity was then calculated using the following equation:

$$\sigma = L/RD \qquad (4)$$

where σ (S cm^{-1}), L (cm), R (Ω), and D (cm) are the proton conductivity, distance between the two electrodes, resistance of the PA-doped membrane, and thickness of the PA-doped membrane, respectively.

The fuel cell performance was measured using single-cell stacks. The pristine membranes were immersed in 85% PA at 160 °C for 24 h, and their ADL was consistent with the above test. The PA-doped membranes and electrode were cut into square samples (membrane: 3.5 × 3.5 cm; electrode: 2.25 × 2.25 cm). The active area of the membrane-electrode assembly was 5 cm^2. The loading of Pt in the catalyst layer was ~1 mg/cm^2.The polarization

curves were obtained without gas humidification at 160 °C using hydrogen (80 mL min^{-1}) and oxygen (160 mL min^{-1}).
